# SARS-CoV-2 S protein disrupts the formation of ISGF3 complex through conserved S2 subunit to antagonize type I interferon response

**DOI:** 10.1128/jvi.01516-24

**Published:** 2024-12-19

**Authors:** Zeng Cai, Wenjia Ni, Wenkang Li, Zhixuan Wu, Xiaoqian Yao, Yucheng Zheng, Yongliang Zhao, Weifeng Yuan, Simeng Liang, Qi Wang, Mingliang Tang, Yu Chen, Ke Lan, Li Zhou, Ke Xu

**Affiliations:** 1 State Key Laboratory of Virology, Taikang Center for Life and Medical Sciences, College of Life Sciences, Wuhan University 12390 , Wuhan, China; 2 Institute for Vaccine Research, Animal Biosafety Level 3 Laboratory, Wuhan University Centre for Animal Experiment 620450 , Wuhan, China; 3 Key Laboratory of Non-coding RNA and Drug Discovery at Chengdu Medical College of Sichuan Province, School of Basic Medical Sciences, Chengdu Medical College 74787 , Chengdu, Sichuan, China; 4 Hubei Jiangxia Laboratory, Wuhan, China; Loyola University Chicago - Health Sciences Campus, Maywood, Illinois, USA

**Keywords:** SARS-CoV-2, spike protein, interferon JAK-STAT pathway, ISGF3 complex, interferon-stimulated gene

## Abstract

Viral immunosuppression substantially affects the host immune response of infected patients and the protective efficacy of vaccines. Here, we found that the spike (S) protein, the major vaccine antigen of severe acute respiratory syndrome coronavirus 2 (SARS-CoV-2), strongly suppresses host innate immunity by inhibiting interferon-stimulated gene (ISG) expression through both S1 and S2 subunits. Mechanistically, the S protein inhibited the formation of the classic interferon-stimulated gene factor 3 (ISGF3) complex composed of STAT1, STAT2, and IRF9 by competing with STAT2 for binding to IRF9, thereby impeding the transcription of ISGs. A strong interaction between S and the STAT1/STAT2 proteins further traps the ISGF3 complex in the endoplasmic reticulum and hinders the nuclear translocation of ISGF3. Notably, the interferon-inhibitory mechanism of the S protein was universal among SARS-CoV-2 variants and other human coronaviruses, including SARS-CoV, Middle East respiratory syndrome coronavirus (MERS-CoV), human coronavirus 229E (HCoV-229E), human coronavirus NL63 (HCoV-NL63), and human coronavirus HKU1 (HCoV-HKU1), through the most evolutionarily conserved region of S2 subunit. Taken together, the findings of this study reveal a new mechanism by which the coronavirus S protein attenuates the host antiviral immune response and provides new insights into the proper design of coronavirus S-based vaccines to prevent immunosuppressive effects.

**IMPORTANCE:**

This study unveils a new mechanism by which the severe acute respiratory syndrome coronavirus 2 (SARS-CoV-2) spike (S) protein attenuates the host’s antiviral immune response. The interferon-inhibitory mechanism of the S protein was universal among SARS-CoV-2 variants and other human coronaviruses, including SARS-CoV, MERS-CoV, HCoV-229E, HCoV-NL63, and HCoV-HKU1, through conserved S2 domains. Our study expands the understanding of SARS-CoV-2 and other human coronaviruses in evading antiviral immune strategies, which is very important for the design and optimization of vaccine antigens, thus providing a theoretical basis for human anti-coronavirus immunity and understanding the interaction between the host and coronavirus.

## INTRODUCTION

Severe acute respiratory syndrome coronavirus 2 (SARS-CoV-2) belongs to the betacoronavirus genus in the Coronaviridae family, which encodes 29 proteins, including 4 structural proteins (spike [S], membrane [M], envelope [E], and nucleocapsid [N]), 16 non-structural proteins (NSP1-NSP16), and 9 accessory proteins (ORF3a, ORF3b, ORF6, ORF7a, ORF7b, ORF8, ORF9b, ORF9c, and ORF10) (1–3). Patients infected with SARS-CoV-2 mainly presented with symptoms such as fever, dry cough, and fatigue; however, some asymptomatic infections have also been observed (4). Clinical studies have reported that despite developing potent cytokines and chemokines in coronavirus disease 2019 (COVID-19) patients, SARS-CoV-2 infection does not induce substantial interferon (IFN) production which indicates the lack of an IFN response (5–9). Additionally, plasma IFN-α2 protein levels are significantly lower in critical patients than in those with mild to moderate illness (10, 11). Although multiple SARS-CoV-2 viral proteins, including NSP1, NSP3, NSP6-NSP8, NSP12-NSP14, NSP16, ORF3a, ORF6, ORF7a, ORF7b, ORF8, ORF9b, S, M, and N, have been reported to suppress cellular innate immunity by inhibiting type I IFN (IFN-I) production and downstream signaling (12–21), the exclusive modulation of immune responses by membrane proteins, notably the S protein as a key antigenic component for vaccine development, needs further exploration.

The IFN-I response is the first line of host defense against invading viruses. The IFN-I response comprises two main facets: the generation of IFNs and the subsequent cellular response to IFN. During the initiation of IFN production, pattern recognition receptors expressed by innate immune cells detect pathogen-associated molecular patterns of viruses (22, 23). RNA viruses such as coronaviruses are recognized by cytoplasmic and endosomal RNA sensors, including RLRs (RIG-I and MDA5) and TLRs (TLR3, TLR7, and TLR8), respectively (24–26). Recognition of RNA viruses by TLRs and RLRs results in the activation of various transcription factors, such as nuclear factor-kappa light-chain enhancer (NF-κB) and IFN regulatory factor 3 (IRF3), leading to translocation to the nucleus and the induction of proinflammatory cytokine, chemokine, and IFN-I expression (27, 28).

In the subsequent IFN response step, IFN α/β activates the Janus kinase (JAK)-signal transducer and transcriptional activator (STAT) signaling pathway via the IFN receptor to stimulate IFN response signaling. In the JAK-STAT signaling cascade, JAK1 and TYK2 mediate the phosphorylation of STAT1 and STAT2 to form a heterodimer, which further binds to IRF9 to form an IFN-stimulating gene factor 3 (ISGF3) complex (29, 30). The ISGF3 complex then translocates into the nucleus, where it initiates the transcription of more than 300 IFN-stimulated genes (ISGs), including IFN-induced transmembrane proteins (IFITMs) 1, 2, and 3, which restrict coronavirus infection (31, 32).

The SARS-CoV-2 S protein comprises two subunits, S1 and S2. The highly mutable S1 subunit mediates viral binding to the host receptor angiotensin-converting enzyme 2 (ACE2), while evolutionarily conserved S2 mediates viral cell membrane fusion (33). Recent studies have shown that the S1 subunit inhibits IFN-α downstream responses through interaction with STAT1 to block its association with JAK1 (21). In this study, we further investigated the crosstalk between the S protein and host IFN responses and discovered that the S2 subunit also played an important role in inhibiting the IFN signaling pathway. The results demonstrated that the S protein, which is located in the endoplasmic reticulum (ER), inhibits IFN-activated downstream signaling. Specifically, the S protein interacts with STAT1, STAT2, and IRF9 through the S2 (aa 688–1273) domain, disrupting the STAT2-IRF9 interaction and thus inhibiting the formation of ISGF3 and sequestering the ISGF3 complex within the ER, which ultimately suppresses downstream ISG production. Given the structural similarities of coronaviruses, we analyzed the function of S proteins, including those from six variants of SARS-CoV-2, as well as the SARS-CoV, MERS-CoV, HCoV-229E, HCoV-NL63, and HCoV-HKU1 viruses. We found that the S2 domain of the aforementioned coronavirus S proteins exhibited high conservation, corresponding to the conserved functionality of inhibiting the IFN-I signaling pathway. Our study introduces a broad-spectrum coronavirus strategy to explore the S proteins that counteract the host IFN response, indicating the need for the optimization of S-based vaccine antigens.

## RESULTS

### The coronavirus S protein antagonizes IFN-stimulated response element activation and IFN-I-dependent ISG Induction

**Fig 1:**
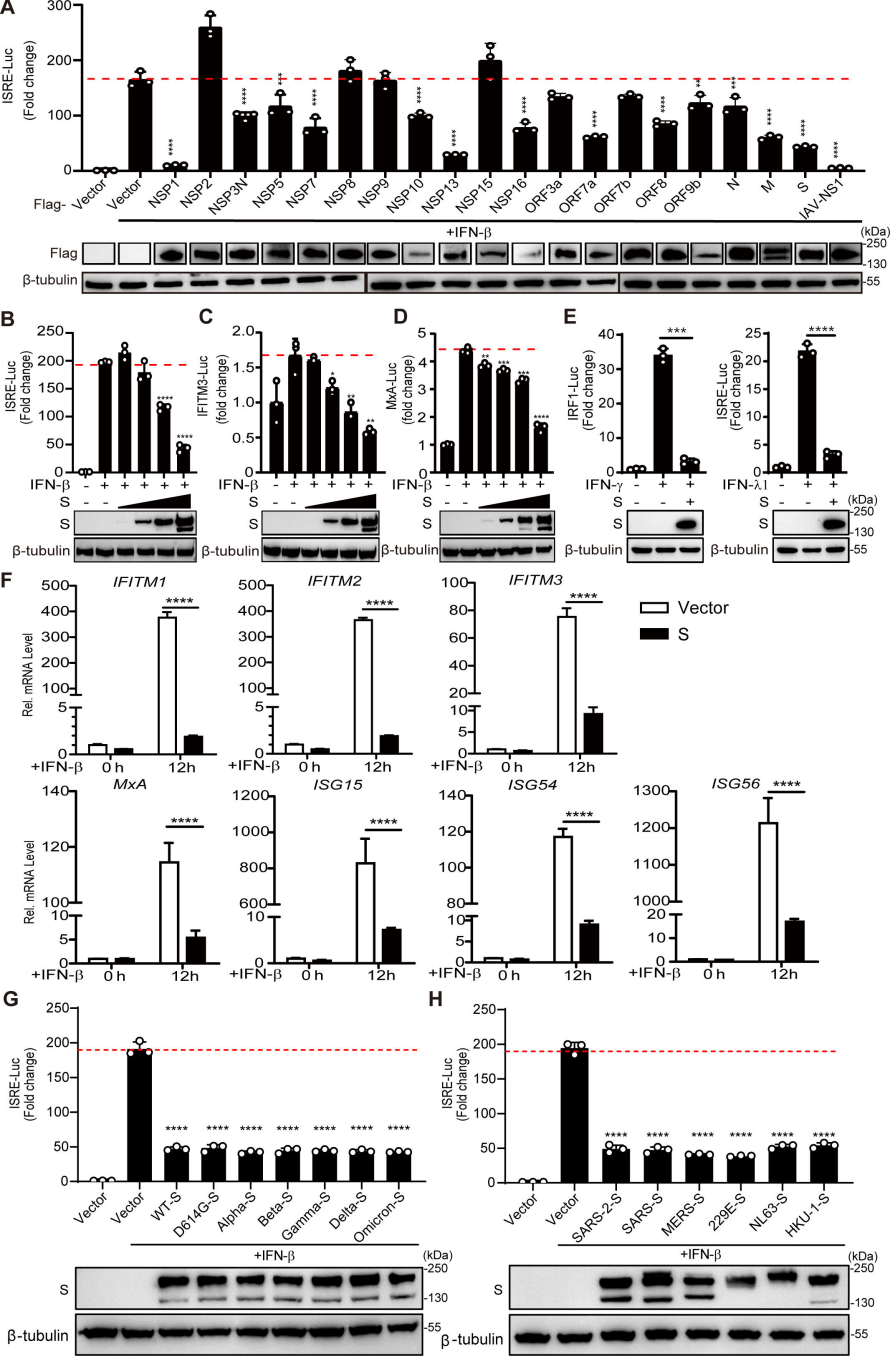
SARS-CoV-2 S proteins inhibit the activation of ISRE and the induction of ISGs. (**A**) HEK293T cells were co-transfected with pISRE-Luc, Renilla luciferase control plasmid pRL-SV40, and SARS-CoV-2 protein-expressing plasmid. Twenty-four hours after the initial transfection, the cells were stimulated with IFN-β (1,000 U/mL) for 12 h, and the luciferase assays were measured. (B, C, and D) HEK293T cells were co-transfected with pISRE-Luc, pIFITM3-Luc, or pMxA-Luc together with the pRL-SV40 plasmid and the plasmid pCAGGS-Flag-S of 0, 0.01, 0.05, 0.25, and 1.25 µg. All promoter activity was measured upon IFN-β (1,000 U/mL) stimulated for 12 h. (**E**) HEK293T cells grown in 24-well plates were co-transfected with pIRF1-Luc or pISRE-Luc and pRL-SV40 control plasmid and a plasmid expressing different S domain proteins. At 24 hours post transfection (hpt), cells were treated with IFN-γ or IFN-λ1 (1,000 U/mL) for 12 h, and the luciferase activity was measured. (**F**) HeLa cells were transfected with empty vector or SARS-CoV-2 S protein-expressing plasmids for 24 h and then stimulated with IFN-β (1,000 U/mL) for 12 h. mRNA expression levels of IFITM1, IFITM2, IFITM3, MxA, ISG15, ISG54, and ISG56 in the collected cells were detected by qRT-PCR. (**G**) HEK293T cells grown in 24-well plates were co-transfected with pISRE-Luc, pRL-SV40 control plasmid, and a plasmid expressing different SARS-CoV-2 variants S proteins. At 24 hpt, cells were treated with IFN-β (1,000 U/mL) for 12 h, and the luciferase activity was measured. (**H**) HEK293T cells grown in 24-well plates were co-transfected with pISRE-Luc, pRL-SV40 control plasmid, and a plasmid expressing SARS-CoV-2 wild type and variants or different coronavirus S proteins. At 24 hpt, cells were treated with IFN-β (1,000 U/mL) for 12 h, and the luciferase activity was measured. Error bars represent mean ± SD from three independent experiments. Statistical significance was determined by the one-way analysis of variance (ANOVA) test. *P < 0.05, **P < 0.01, ***P < 0.001, and ****P < 0.0001.

Previous reports have shown that SARS-CoV-2 infection does not induce an effective IFN-I response in COVID-19 patients, especially in critical patients (5–9). We hypothesized that multiple proteins of SARS-CoV-2 are involved in the regulation of the IFN-I signaling pathway. To test this hypothesis, we cloned 29 genes of SARS-CoV-2 after codon optimization and successfully expressed 19 proteins: 11 nonstructural proteins, 3 structural proteins, and 5 accessory proteins. We screened the genes by cotransfection of the plasmid encoding IFN-stimulated response element (ISRE) and the internal control plasmid pRL-SV40 into HEK293T cells, followed by a luciferase reporter assay. In addition, an NS1-expressing plasmid for influenza virus (A/WSN/33, H1N1) was used as a positive control (Fig. 1A). As expected, the positive control (NS1) and the NSP1, NSP3, NSP13, NSP16, ORF3a, ORF7a, ORF7b, ORF8, S, M, and N proteins exhibited ISRE inhibitory effects, as reported in previous articles (12–21, 34). Unexpectedly, we also found that the S, NSP5, NSP7, NSP10, NSP16, and ORF9b proteins significantly inhibited ISRE activation. As the S protein acts as a structural protein to mediate viral invasion and serves as a vaccine antigen, we focused on the role of the S protein in inhibiting ISRE activity in subsequent studies. A dose-dependent assay revealed that the S protein strongly inhibited IFN-I-induced activation of the ISRE, IFITM3, and MxA reporters (Fig. 1B through D). Moreover, the S protein could also inhibit IFN-γ-induced activation of the IRF1 reporter and IFN-λ-induced activation of the ISRE reporter (Fig. 1E). Next, we overexpressed the S protein and examined the endogenous expression of IFITM1, IFITM2, IFITM3, MxA, ISG15, ISG54, and ISG56 induced by IFN-I through quantitative PCR with reverse transcription (qRT-PCR) analysis. We observed that the expression of these ISG genes was significantly inhibited ~200-, 192-, 8-, 20-, 115-, 13-, and 72-fold, respectively (Fig. 1F). Notably, we found that the S proteins of the D614G variant, Alpha variant (B.1.1.7), Beta variant (B.1.351), Gamma variant (P.1), Delta variant (B.1.617.2), and Omicron variant (B.1.1.529) significantly inhibited IFN-I-mediated ISRE activation, and their inhibitory effect was not significantly different from that of the wild-type virus (Fig. 1G). When we compared the S proteins of other human coronaviruses, including SARS-CoV-2, SARS-CoV, MERS-CoV, HCoV-229E, HCoV-NL63, and HCoV-HKU1, equivalent inhibitory effects on ISRE promoter activation were found among all the tested S proteins (Fig. 1H), indicating that the S protein of coronaviruses plays a conserved role in suppressing the host IFN response pathway.

### The SARS-CoV-2 S protein antagonizes the JAK-STAT signaling pathway by targeting ISGF3

**Fig 2:**
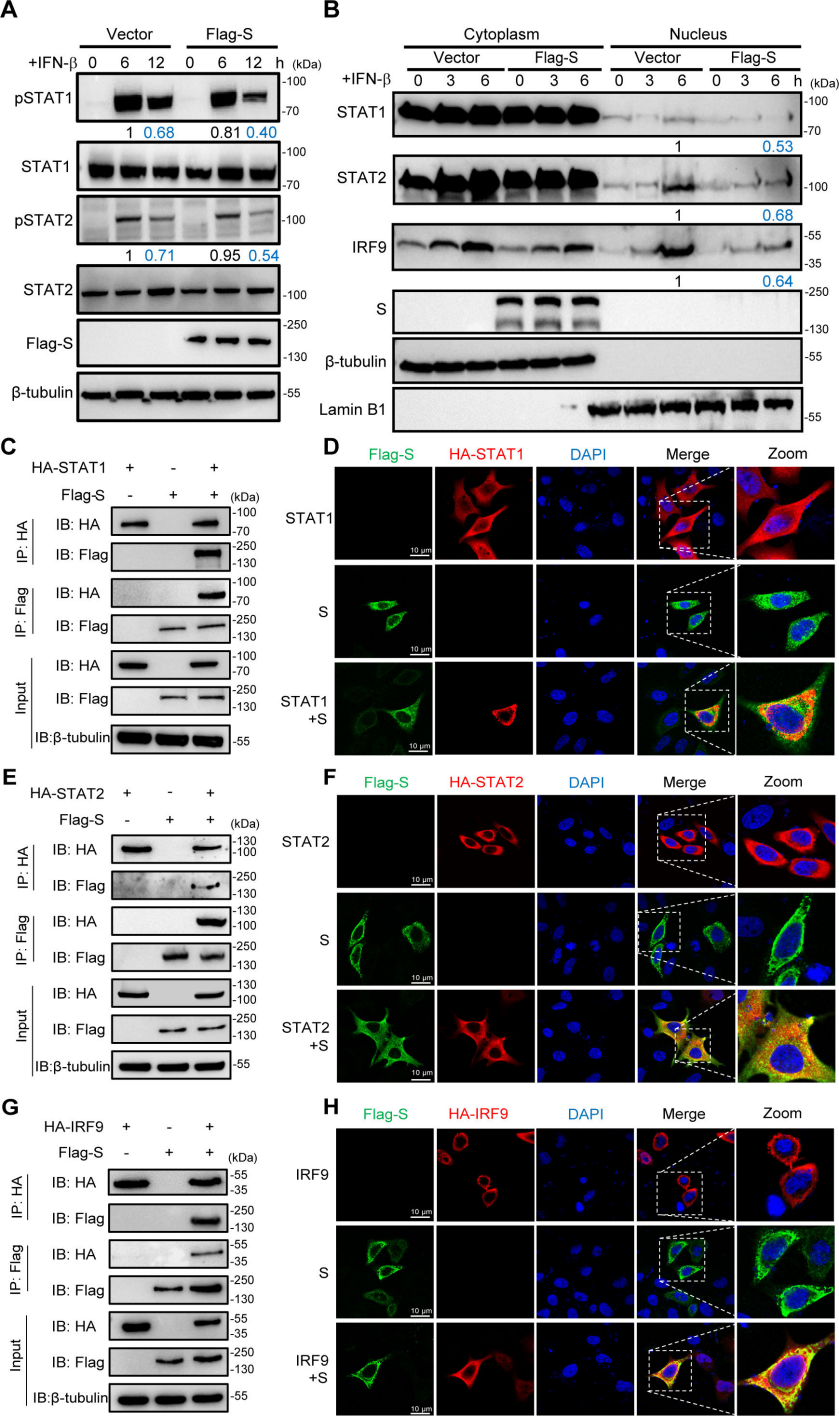
SARS-CoV-2 S protein inhibits phosphorylation of STAT1/STAT2 and blocks STAT1 nuclear translocation. (**A**) HeLa cells were transfected with empty vector plasmid or SARS-CoV-2 S protein-expressing plasmids for 24 h and then stimulated with IFN-β for 6 or 12 h and analyzed by western blot using anti-pSTAT1, anti-total STAT1, anti-pSTAT2, and anti-total STAT2 antibodies. Protein band intensity was quantitated using ImageJ software. Data represent the means of three independent experiments. (**B**) HeLa cells were transfected with empty vector plasmid or SARS-CoV-2 S protein-expressing plasmids. After 24 h, the cells were stimulated with IFN-β for 3 or 6 h and fractionated into cytoplasmic and nuclear fractions. The fractions were analyzed by western blotting for STAT1, STAT2, and IRF9 detection. β-Tubulin and Lamin B1 were used as cytoplasmic and nuclear markers, respectively. (C, E, and G) HEK293T cells were transfected with the plasmid as indicated. At 48 hpt, the cells were harvested and subjected to a Co-IP assay with the anti-HA magnetic beads. β-Tubulin was shown as a loading control (input). (D, F, and H) HeLa cells were transfected with plasmid expression HA-STAT1, HA-STAT2, or HA-IRF9 along with Flag-S for 24 h. Cells were fixed and permeabilized, staining with anti-HA and anti-FLAG as primary antibodies and anti-Alexa Fluor 488, and anti-Alexa Fluor 568 as secondary antibodies. Scale bar, 10 µm.

To investigate the mechanisms by which the SARS-CoV-2 S protein antagonizes the IFN-β downstream signaling pathway, we examined the phosphorylation of STAT1/STAT2 in the JAK/STAT pathway in response to IFN-β stimulation. Thus, HeLa cells were transiently transfected with the S protein or empty vector plasmid followed by treatment with IFN-β for 6 h or 12 h, after which the expression and phosphorylation levels of STAT1/STAT2 were measured by western blotting. As expected, overexpression of the SARS-CoV-2 S protein attenuated the phosphorylation of STAT1 and STAT2 (Fig. 2A). Once STAT1/STAT2 is phosphorylated by IFN-β stimulation, pSTAT1 and pSTAT2 form a heterodimer, which recruits IRF9 to form the STAT1/STAT2/IRF9 complex (ISGF3). Then, ISGF3 translocates to the nucleus and binds to the ISRE, thereby leading to the expression of ISGs. Thus, we determined the effect of the SARS-CoV-2 S protein on IFN-β-induced ISGF3 nuclear translocation. HeLa cells transiently expressed the S protein or empty vector plasmid and were subsequently treated with IFN-β for 3 h or 6 h. Western blotting showed that ISGF3 nuclear translocation was disrupted by the overexpression of the S protein (Fig. 2B). Since the S protein inhibits the phosphorylation of STAT1/STAT2 and suppresses ISGF3 nuclear translocation, we next examined whether the S protein can interact with STAT1, STAT2, and IRF9, which are components of the ISGF3 complex. We cotransfected plasmids encoding the S protein with STAT1, STAT2, or IRF9, followed by coimmunoprecipitation (Co-IP). As shown in Fig. 2C, E and G, the Flag-tagged S protein precipitated with HA-tagged STAT1, STAT2, and IRF9. Immunofluorescence data showed that the S protein colocalized with STAT1, STAT2, and IRF9 (Fig. 2D, F and H). These results suggest that the S protein not only inhibits the phosphorylation of STAT1 and STAT2 but also binds to ISGF3 components.

### The SARS-CoV-2 S protein targets and anchors ISGF3 to the ER

**Fig 3:**
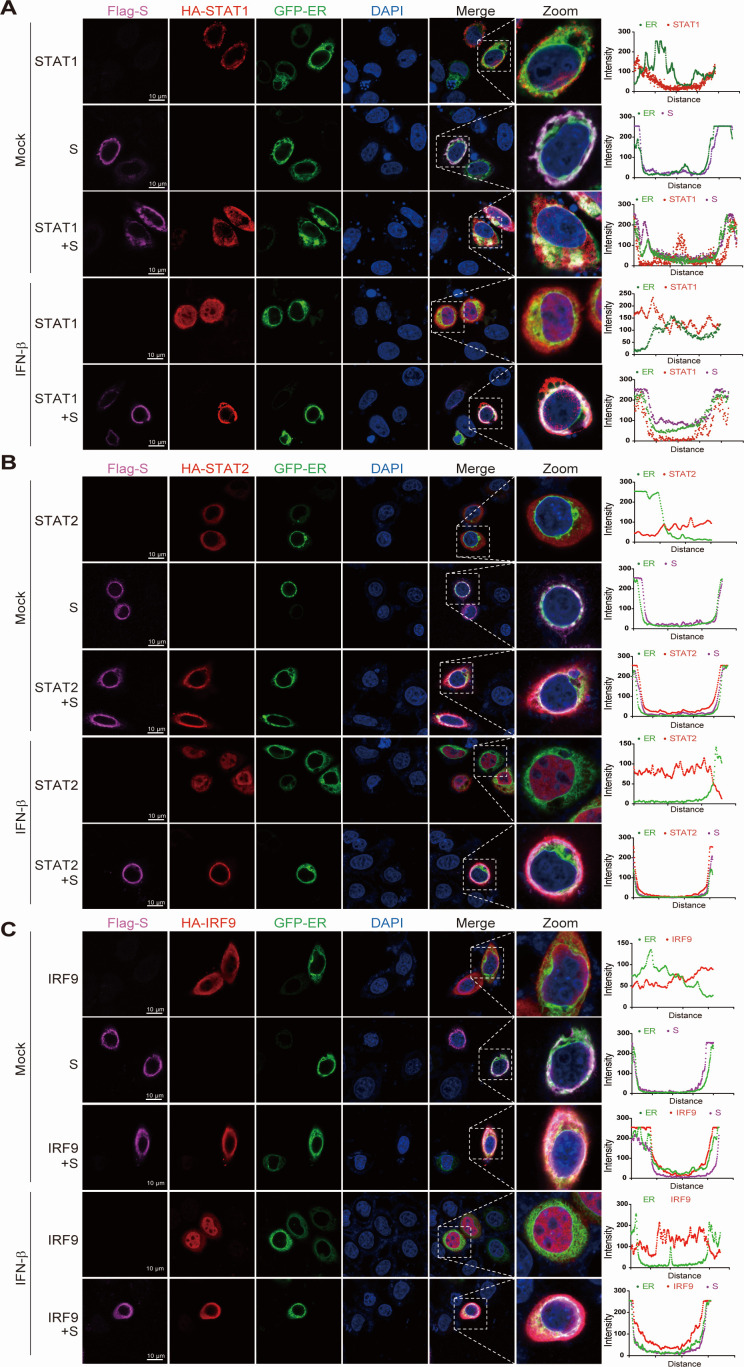
SARS-CoV-2 S strapped ISGF3 on ER. (**A**) HeLa cells were transfected with plasmid expression HA-STAT1, GFP-Sec61β (ER marker), and Flag-S for 24 h and then treated with IFN-β (1,000 U/mL) for 30 min. Cells were fixed and permeabilized, staining with anti-HA and anti-FLAG as primary antibodies and anti-Alexa Fluor 568 and anti-Cy5 as secondary antibodies. (**B**) HeLa cells were transfected with plasmid expression HA-STAT2, GFP-Sec61β (ER marker), and Flag-S for 24 h and then treated with IFN-β (1,000 U/mL) for 30 min. Cells were fixed and permeabilized, staining with anti-HA and anti-FLAG as primary antibodies and anti-Alexa Fluor 568 and anti-Cy5 as secondary antibodies. (**C**) HeLa cells were transfected with plasmid expression HA-IRF9, GFP-Sec61β (ER marker), and Flag-S for 24 h and then treated with IFN-β (1,000 U/mL) for 30 min. Cells were fixed and permeabilized, staining with anti-HA and anti-FLAG as primary antibodies and anti-Alexa Fluor 568 and anti-Cy5 as secondary antibodies. Scale bar, 10 µm.

To further explore the subcellular colocalization of the S protein with STAT1, STAT2, and IRF9, we transiently transduced GFP-Sec61b (ER markers) with the vector or S protein plasmid into HeLa cells. As depicted in Fig. 3A through C, STAT1, STAT2, and IRF9 were initially distributed in the cytoplasm in the resting state, while the S protein was found in the ER. However, after transfection with the S protein, STAT1, STAT2, and IRF9 colocalized with the ER. Upon stimulation with IFN-I, substantial nuclear translocation of individually transfected STAT1, STAT2, and IRF9 was observed. However, when cells were cotransfected with the S protein, the nuclear translocation of STAT1, STAT2, and IRF9 was significantly inhibited, and these proteins colocalized with the S protein anchored on the ER. This finding indicates that the S protein anchored to the ER significantly inhibits the nuclear translocation of the activated ISGF3 complex by interacting with STAT1, STAT2, and IRF9. Taken together, these data revealed that the S protein interacted with STAT1, STAT2, and IRF9 and anchored them to the ER to prevent the nuclear translocation of ISGF3.

### The SARS-CoV-2 S protein interferes with the formation of the ISGF3 complex

**Fig 4:**
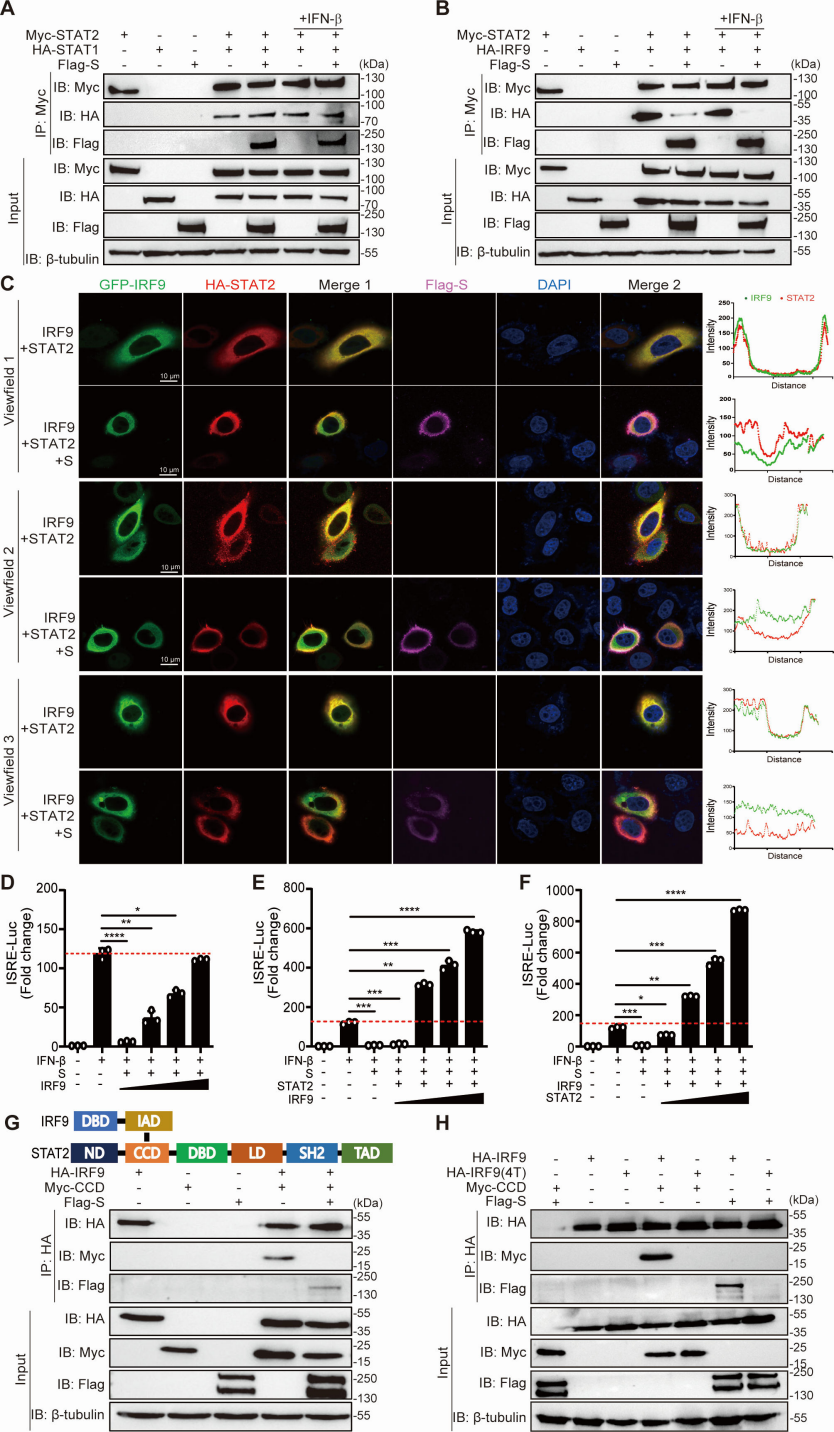
SARS-CoV-2 S interferes with the formation of the STAT2-IRF9 cognate complex. (**A, B**) HEK293T cells were transfected with the plasmid as indicated for 48 h and then treated with IFN-β (1,000 U/mL) for 1 h. Cells were harvested and subjected to a Co-IP assay with the anti-Myc magnetic beads. β-Tubulin was shown as a loading control (input). (**C**) HeLa cells were transfected with plasmid expression HA-STAT2, GFP-IRF9, and Flag-S for 24 h. Cells were fixed and permeabilized, staining with anti-HA and anti-FLAG as primary antibodies and anti-Alexa Fluor 568 and anti-Cy5 as secondary antibodies. Scale bar, 10 µm. (**D**) HEK293T cells grown in 24-well plates were co-transfected with pISRE-Luc, pRL-SV40 control plasmid, a plasmid expressing different S domain proteins, and a plasmid expressing IRF9 (0 ng, 50 ng, 250 ng, and 1,250 ng). At 24 hpt, cells were treated with IFN-β (1,000 U/mL) for 12 h, and the luciferase activity was measured. (**E**) HEK293T cells grown in 24-well plates were co-transfected with pISRE-Luc, pRL-SV40 control plasmid, a plasmid expressing S, a plasmid expressing STAT2 protein, and a plasmid expressing IRF9 (0 ng, 50 ng, 250 ng, and 1,250 ng). At 24 hpt, cells were treated with IFN-β (1,000 U/mL) for 12 h, and the luciferase activity was measured. (**F**) HEK293T cells grown in 24-well plates were co-transfected with pISRE-Luc, pRL-SV40 control plasmid, a plasmid expressing S, a plasmid expressing IRF9 protein, and a plasmid expressing STAT2 (0 ng, 50 ng, 250 ng, and 1,250 ng). At 24 hpt, cells were treated with IFN-β (1,000 U/mL) for 12 h, and the luciferase activity was measured. (**G**) Domain organization of STAT2 and IRF9. CCD, coiled-coil domain; DBD, DNA-binding domain; IAD, IRF-association domain; LD, linker domain; ND, N-domain; SH2, Src homology domain; TAD, transactivation domain. HEK293T cells were transfected with the plasmid as indicated for 48 h. Co-IP was performed with anti-HA magnetic beads. β-Tubulin was shown as a loading control (input). (**H**) HEK293T cells were transfected with the plasmid-encoded CCD, S protein, and IRF9 wild-type or mutant plasmid (4T), containing L233A, R236E, L274A, and F283A, for 48 h. Co-IP was performed with anti-HA magnetic beads. β-Tubulin was shown as a loading control (input). Error bars represent mean ± SD from three independent experiments. Statistical significance was determined by the one-way ANOVA test. *P < 0.05, **P < 0.01, ***P < 0.001, and ****P < 0.0001.

Because the formation of ISGF3 is a crucial step in the expression of ISGs, we next investigated whether the S protein affects the formation of the STAT1-STAT2 and STAT2-IRF9 complexes, which are essential for ISGF3 activation. We cotransfected plasmids expressing STAT2 and S with the STAT1 or IRF9 protein, followed by Co-IP. The results demonstrated that while the interaction between STAT1 and STAT2 remained unaffected by the S protein (Fig. 4A), the S protein strongly inhibited the binding of STAT2 to IRF9 under the same conditions (Fig. 4B). These results were further supported by the results of the immunofluorescence assay, which showed that in the absence of the S protein, STAT2 and IRF9 were completely colocalized, whereas this colocalization was largely disrupted in the presence of the S protein (Fig. 4C). These results demonstrated that the S protein inhibited the recruitment of IRF9 by the STAT1/STAT2 complex. Subsequently, we hypothesized that dose-expressing IRF9 in cells that the S protein also expressed could potentially reverse the inhibitory impact of S protein on ISGF3 complex formation. To test the hypothesis, S protein was overexpressed in HEK293T cells and IRF9 was expressed at increasing levels. Results from the experiments indicated that increasing the transfection amount of IRF9 can fully restore the inhibitory effect of S protein on IFN-I-mediated ISRE promoter activation (Fig. 4D). Moreover, when cells were co-transfected with S protein and low-level IRF9 alongside an increasing dose expression of STAT2 or with S protein and low-level STAT2 alongside an increasing dose expression of IRF9, it resulted in strong activation of the ISRE promoter induced by IFN-I (Fig. 4E and F). These data indicated that the S protein does not affect the interaction between STAT1 and STAT2 but rather interacts with IRF9, thereby hindering the recruitment of IRF9 by STAT2.

Previous studies revealed that STAT2 interacts with IRF9 through its coil-coiled domain (CCD) (35). To determine whether the presence of S affects the association of STAT2-CCD with IRF9, we carried out Co-IP assays and found that S protein expression led to a notable decrease in the interaction between IRF9 and STAT2-CCD (Fig. 4G). Thus, the S protein competes with STAT2-CCD for binding with IRF9. To further determine the functional sites of the S protein that interfere with the formation of the STAT2-IRF9 complex, we constructed an IRF9 mutant plasmid (4T) containing L233A, R236E, L274A, and F283A, which are located in the C-terminal IRF-association domain (IAD) and have been reported to eliminate the interaction between IRF9 and STAT2 (35). Our results showed that while IRF9-WT was able to robustly coimmunoprecipitate with S or STAT2-CCD, the interaction between IRF9-WT and IRF9-(4T) was reduced to background levels. In brief, both STAT2 and S interact with IRF9 through the same domain on IRF9, wherein STAT2 and S compete with each other to bind to IRF9 (Fig. 4H). Together, our findings indicated that the SARS-CoV-2 S protein disturbs the formation of the ISGF3 complex, which in turn suppresses the JAK/STAT signaling pathway.

### The S2 domain plays a crucial role in the inhibitory function of ISGF3

**Fig 5:**
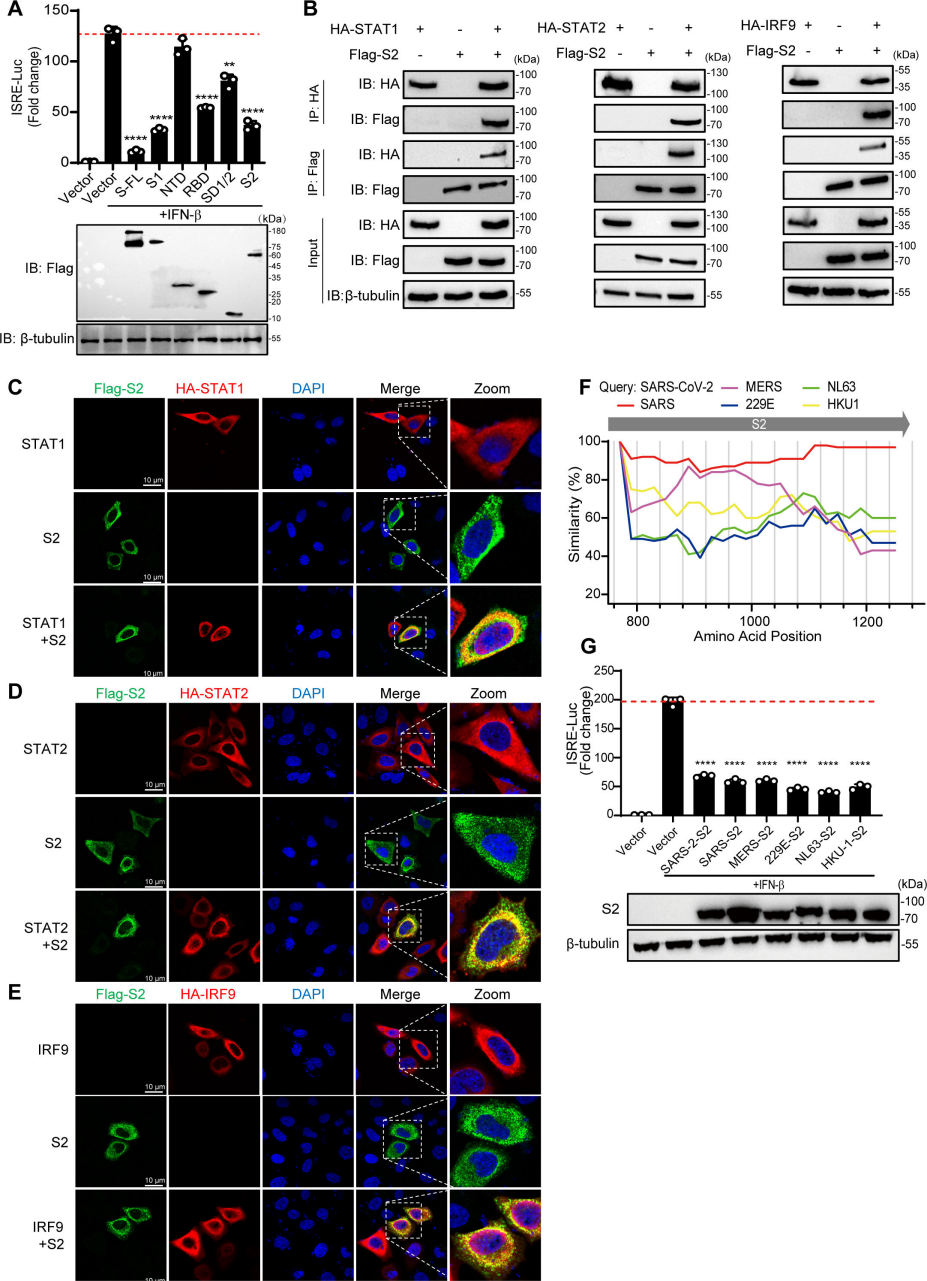
The S2 domain is mainly responsible for the inhibition mechanism. (A) HEK293T cells grown in 24-well plates were co-transfected with pISRE-Luc, pRL-SV40 control plasmid, and a plasmid expressing different S domain proteins. At 24 hpt, cells were treated with IFN-β (1,000 U/mL) for 12 h, and the luciferase activity was measured. FL, full length. (B) HEK293T cells were transfected with the plasmid as indicated. At 48 hpt, the cells were harvested and subjected to a Co-IP assay with the anti-HA magnetic beads. β-Tubulin was shown as a loading control (input). (C, D, and E) HeLa cells were transfected with a plasmid expressing HA-STAT1, HA-STAT2, or HA-IRF9 along with Flag-S2 for 24 h. Cells were fixed and permeabilized, staining with anti-HA and anti-FLAG as primary antibodies and anti-Alexa Fluor 488 and anti-Alexa Fluor 568 as secondary antibodies. Scale bar, 10 µm. (F) Simplot analyses the similarity of different human coronavirus S protein sequences. The abscissa represents the amino acid position of the SARS-CoV-2 S protein. (G) HEK293T cells grown in 24-well plates were co-transfected with pISRE-Luc, pRL-SV40 control plasmid, and a plasmid expressing different coronavirus S2 domains. At 24 hpt, cells were treated with IFN-β (1,000 U/mL) for 12 h, and the luciferase activity was measured. Error bars represent mean ± SD from three independent experiments. Statistical significance was determined by the one-way ANOVA test. **P < 0.01 and ****P < 0.0001.

To determine the pivotal domain involved in the inhibitory effect of the S protein on the JAK/STAT signaling pathway, we divided the full-length S protein into five domains: S1 (aa 12–680), N-terminal domain (NTD) (aa 12–306), RBD (aa 328–533), SD1/2 (aa 535–680), and S2 (aa 688–1273). HEK293T cells were transfected with plasmids expressing the five domains, pISRE-Luc or pRL-SV40, followed by a dual-luciferase assay. The results showed that the S protein inhibited the activity of ISRE by ~91%, whereas the S1 and S2 domains inhibited the activity of ISRE by ~70%. However, the NTD did not affect the activity of ISRE triggered by IFN-β. The RBD and SD1/2 only slightly inhibited the activity of ISRE by ~57% and ~36%, respectively (Fig. 5A). These data indicated that the S1 and S2 domains play an important role in inhibiting the activity of the ISRE promoter. As the S2 domain is more conserved among coronaviruses and had not been elucidated as a IFN antagonist, we focused on S2 and verified that the S2 domain can interact with STAT1, STAT2, and IRF9 (Fig. 5B), and the immunofluorescence results confirmed these findings (Fig. 5C through E). Moreover, the S2 domain suppressed STAT1 nuclear translocation induced by IFN-β (data not shown). We therefore speculate that other coronaviruses also inhibit the IFN response pathway through the S2 region. We aligned different human coronavirus S protein sequences, including those of SARS-CoV-2, SARS-CoV, MERS-CoV, HCoV-229E, HCoV-NL63, and HCoV-HKU1, and analyzed them with Simplot software. The S2 domain was highly similar among different human coronaviruses (Fig. 5F). As expected, the S2 domain of all tested coronavirus S proteins exerted a similar inhibitory effect on the activity of the ISRE reporter stimulated by IFN-β (Fig. 5G). Taken together, our data highlight the critical role of the S2 region as a conserved domain in coronaviruses that effectively inhibits the IFN-I pathway.

## DISCUSSION

**Fig 6:**
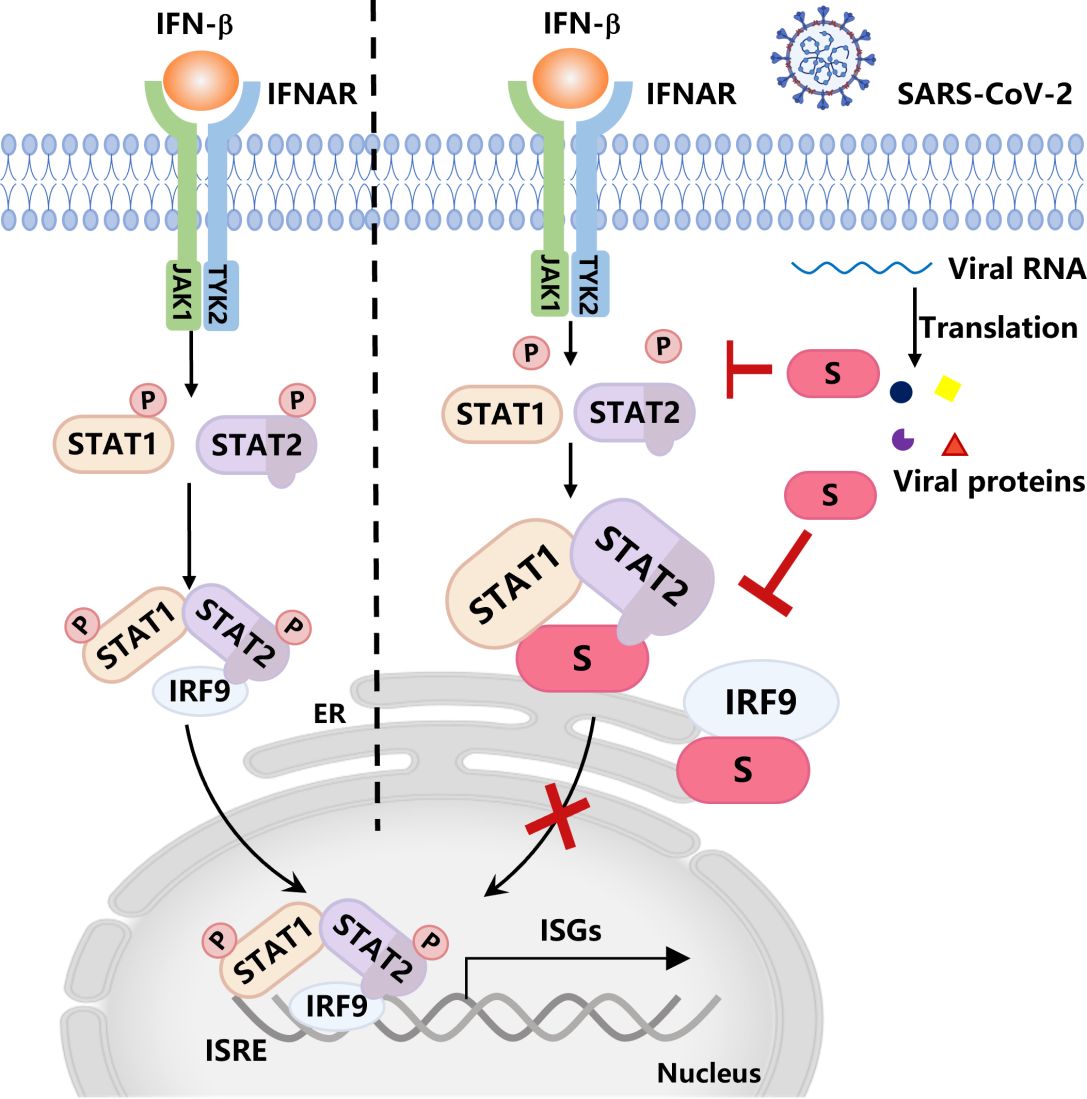
Working model SARS-CoV-2 S protein blocks IFN-β signaling by impeding the phosphorylation of STAT1/STAT2 and disrupting the STAT2-IRF9 cognate complex.

Immunosuppressive conditions strongly affect the immune response of vaccine recipients, resulting in a relatively suboptimal immune response after vaccination. Clinical studies have shown that the decline in immunity to SARS-CoV-2 starts within the first month following complete vaccination, persisting until the sixth month, at which point the immunity level may not offer sufficient protection against SARS-CoV-2 (36–41). In contrast, long-term studies of antibody responses to vaccinia, measles, mumps, or rubella suggest that these responses generally stabilize with half-lives > 10 years (42, 43). An increasing number of studies have suggested that SARS-CoV-2 may develop various strategies to limit effective IFN production, and the NSP1, NSP3, NSP6, NSP7, NSP8, NSP12, NSP13, NSP14, NSP16, ORF3a, ORF6, ORF7a, ORF7b, ORF8, ORF9b, M, S, and N proteins have been previously reported to inhibit both IFN-I production and downstream signaling (12–21, 34, 44, 45). For example, ORF6 antagonizes the IFN-I response via its C-terminus and inhibits STAT1 nuclear translocation but not phosphorylation (12); another study reported that ORF6 hijacks Nup98 to block STAT nuclear import (15). M has been shown to affect RIG-I and TRIM25 to inhibit NF-κB signaling, impairing the phosphorylation of STAT1 and the nuclear translocation of STAT1 (46). N suppresses the phosphorylation and nuclear translocation of STAT1 and STAT2 (14). The S protein (S protein) serves as a pivotal factor for the cellular entry of SARS-CoV-2 and is a key antigenic component in COVID-19 vaccines (47). The substantial decline in the protective efficacy of COVID-19 vaccines indicates that the S protein may be involved in suppressing cellular immune responses. Here, we reported that the coronaviral S protein can target the JAK-STAT pathway to block the production of downstream ISGs, especially by disrupting the ISGF3 complex to inhibit the host antiviral immune signaling pathway (Fig. 6).

The SARS-CoV-2 S protein is composed of two subunits, namely, S1 and S2, which play different roles in the viral infection process. S1 contains the receptor-binding domain responsible for binding to the receptor on the host cell (48, 49), while S2 contains the fusion peptide required for viral entry, mediating virus-cell membrane fusion or entry into cells via endocytosis, making it one of the prime targets for antiviral drug design (50, 51). Despite the continuous transmission and emergence of new variants of SARS-CoV-2, the mutation sites of the S protein in the variants are mostly concentrated in the S1 domain. These amino acid mutations alter the infectivity of the virus, leading to the evasion of neutralizing antibodies, while the S2 domain remains relatively conserved among different SARS-CoV-2 mutant strains (52, 53). In this study, we found that the S2 subunit plays a critical role in attenuating the IFN signaling pathway. Interestingly, our data showed that the S proteins of SARS-CoV-2 (variants of concern) showed inhibition of ISRE activity similar to that of the wild-type strains. Moreover, our analysis indicated that the S2 domain was highly similar among different human coronaviruses. Consistent with our expectations, the S2 domains of SARS-CoV, MERS-CoV, HCoV-229E, HCoV-NL63, and HCoV-HKU-1 also inhibited the IFN-β-induced ISRE activity (Fig. 5F and G). These results suggested that the ability of coronaviral S proteins to inhibit the formation of ISGF3 through the S2 domain and thus suppress the activation of the IFN-I signaling pathway is conserved.

The IFN response is the first line of defense against invading viruses during viral infection. Previous studies have shown that severe attenuation of IFN downstream cytokine expression is observed in patients with severe symptoms of SARS-CoV-2 infection (7–9). For example, IFN-I and ISG56 are barely induced in the early stage of virus infection but increase in the late stage. This delayed antiviral response may provide a window into the activation and evaluation of IFN-I responses induced by SARS-CoV-2 (5, 6, 12). Exogenous IFN-I has also been used in clinical therapy due to the insufficient production of IFN-I in patients with severe symptoms and asymptomatic-infected individuals (54). However, researchers have also proven that IFN-β does not show significant efficacy in the treatment of COVID-19 (55, 56), indicating that SARS-CoV-2 not only inhibits the production of IFN-I but also inhibits the IFN-I response, and the mechanism underlying inhibition of IFN-I still needs to be studied. Our results indicate that the S protein antagonizes the IFN-I downstream JAK-STAT pathway, partially explaining the attenuation of the IFN-I response in patients with COVID-19. Consistent with the cellular-level experimental findings, the result in a murine model post-SARS-CoV-2 infection confirmed a substantial downregulation of downstream IFN-responsive genes (57). Previous studies have shown that STAT1 is diffusely distributed in the cytoplasm at rest, while STAT2 and IRF9 can form a complex and translocate to the nucleus to maintain low-level expression of ISGs. Under IFN stimulation, STAT1 and STAT2 are phosphorylated, form an ISGF3 complex with IRF9, and enter the nucleus, which is the key to the strong production of ISGs (15, 46, 58). Recent studies have shown that the S protein interacts with STAT1 to block its association with JAK1, and S protein-mediated attenuation of IFN-α downstream responses is dependent mainly on its S1 subunit (21). In our study, we confirmed the result that the S1 subunit attenuated IFN-β downstream responses. Besides, we also found that S2 played a crucial role in the inhibition of IFN-β downstream responses. Compared with the vector, the full-length S protein attenuated IFN-β-induced ISRE reporter activation by approximately 91%, indicating more comprehensive inhibitory effects on IFN downstream signaling. Interestingly, the NTD did not inhibit the activation of the ISRE reporter, whereas the RBD and SD1/2 only resulted in approximately 57% and 36% inhibition, respectively. The S1 subunits and S2 demonstrated a strong inhibitory efficiency of approximately 70%, similar to that of full-length S (Fig. 5A), suggesting that the IFN antagonist ability requires an intact subunit of S proteins. Besides JAK1/STAT1 being inhibitory in STAT1 phosphorylation signaling, we further disclosed that the downstream ISGF3 complex is disrupted and trapped outside the nucleus by S/S2 protein, suggesting an impressive IFN antagonist role of S protein. Moreover, we found that the S2 domain had highly similar IFN antagonist abilities in different human coronaviruses, including SARS-CoV, MERS-CoV, HCoV-229E, HCoV-NL63, and HCoV-HKU1 (Fig. 5F and G). These results indicated that the S2 domain of the S protein of coronavirus also mediated the inhibition of the IFN-I pathway. We mainly investigated IFN-β instead of IFN-α because IFN-α is blocked in the production step, leaving the IFN-β response to be the major target for SARS-CoV-2 to combat (IFN-α production is diminished in mouse model) (59), and the potential for response to IFN-α was not affected in COVID-19 patients (10).

To clarify the interaction between membrane-located S protein with cytoplasmic STAT proteins, we further tested the IFN-antagonist ability of S protein and the S2 domain without the transmembrane motif (TM) domain. The luciferase assay results demonstrated that S_ΔTM_ and S2_ΔTM_ still significantly suppressed the activation of the IFN-β-induced ISRE promoter activity (Fig. S1A). In cells over-expressing STAT1 and S_ΔTM_ or S2_ΔTM_ protein, the S_ΔTM_ and S2_ΔTM_ protein could again interact with STAT1 with or without IFN-β stimulating in both Co-IP assay (Fig. S1B and C) and immunofluorescence collocalization assay (Fig. S1D and E). These data indicated that S protein interacts with STATs probably through its cytoplasmic body domain outside the ER lumen. The orientation of S on the ER membrane is still controversial and needs further studies.

The interaction between STAT2 and the IAD of IRF9 is essential for the formation of ISGF3 and the generation of ISGs (60). The STAT2-IRF9 complex and the NF-κB subunit p65 have been reported to be important elements in activating IL-6 expression (61). Mariani et al. also revealed that the STAT2-IRF9 complex increased the expression of both IFN-β- and TNF-55-stimulating genes (62). Therefore, STAT2-IRF9 and ISGF3 may be important complexes for regulating signaling pathways at the promoter level. Our study showed that the S protein was localized in the ER and could anchor STAT1 as well as STAT2 and IRF9 in the cytoplasm to the ER via interactions, which might reveal the molecular mechanism by which the S protein inhibits the interaction between JAK1 and STAT1. Moreover, the S protein can disrupt the interaction between STAT2 and IRF9 and inhibit the formation of ISGF3, thus inhibiting the IFN signaling pathway. A previous study revealed that IRF9 contains four mutant sites located in the IAD and cannot bind to STAT2, thus disrupting the formation of ISGF3 (35). Interestingly, our study showed that the S protein strongly interacts with IRF9; however, the four mutation sites located in the IAD of IRF9 eliminated the interaction with the S protein. This finding indicated that the S protein attenuated the interaction of STAT2 with IRF9 by competitively binding to IAD and subsequently inhibited the formation of ISGF3. In summary, our data revealed a novel mechanism by which the S protein inhibits the formation of ISGF3 and provided evidence of asymptomatic COVID-19 infection.

Taken together, our study expands the understanding of SARS-CoV-2 and other human coronaviruses in evading antiviral immune strategies, which is very important for the design and optimization of vaccine antigens, thus providing a theoretical basis for human anti-coronavirus immunity and understanding the interaction between the host and coronavirus.

## MATERIALS AND METHODS

### Cell culture

The HEK293T and HeLa cells were obtained from American Type Culture Collection and maintained in Dulbecco’s modified Eagle medium (Gibco) supplemented with 10% fetal bovine serum (FBS) and 1% penicillin and streptomycin (P/S, Gibco) at 37°C in an incubator with 5% CO_2_.

### Quantitative real-time PCR

The mRNA levels of the indicated genes were quantified via qRT-PCR. Total RNA was extracted by using TRIzol (Invitrogen, cat #15596018) and reverse transcript to cDNA by the PrimeScrip RT Reagent Kit (Takara, cat #RR037A). The corresponding cDNAs were quantified by using Hieff qPCR SYBR Green Master Mix (Yeason, cat #11202ES03). The Primer sequences are provided below. All mRNA levels were normalized to the β-actin level or mActb level.

The primers are listed in Table 1.

**TABLE 1:** Primers used in this study

Gene	Forward primer (5′→3′)	Reverse primer (5′→3′)
*IFITM1*	CCAAGGTCCACCGTGATTAAC	ACCAGTTCAAGAAGAGGGTGTT
*IFITM2*	ATGAACCACATTGTGCAAACCT	CCCAGCATAGCCACTTCCT
*IFITM3*	CACCTCCTCCCCTTCCTCA	TGTCCCCGGCTATCCACTA
*ISG15*	CCTCCAGCCCGCTCACTTGC	AGGACAGGGTCCCCCTTGCC
*ISG54*	CTGCAACCATGAGTGAGAA	CCTTTGAGGTGCTTTAGATAG
*ISG56*	TACAGCAACCATGAGTACAA	TCAGGTGTTTCACATAGGC
*IFN-γ*	TCGGTAACTGACTTGAATGTCCA	TCGCTTCCCTGTTTTAGCTGC
*MxA*	GATCATGTGCTGAATGCCTAGCAC	GTGGAGCCTGACCTTGTGGCACTG
*β-actin*	CATGTACGTTGCTATCCAGGC	CTCCTTAATGTCACGCACGAT

### Plasmid and transfection

The plasmid-encoding SARS-CoV-2 ORFs were cloned into the pCAGGS vector. The plasmid encoding SARS-CoV-2 S protein with Flag-tag was purchased from SinoBiological (cat #VG40589-NF). Mammalian expression plasmids for HA-, Flag-, or myc-tagged STAT1, STAT2, and IRF9 were constructed by standard molecular biology techniques. pISRE-Luc and pRL-SV40 were preserved in our laboratory. The S protein of SARS-CoV-2 variants (Alpha, Beta, Gamma, and Delta), SARS-CoV, MERS-CoV, 229E, NL63, HKU-1, and S1, NTD, RBD, SD1&SD2, and S2 of SARS-CoV-2 S protein was cloned into the pCAGGS vector. The DNA transfection reagent using Lipofectamine 2000 (Invitrogen) and conducted according to the manufacturer’s protocol.

### Luciferase assay

HEK293T cells were seeded in 24-well plates and were transfected with a control plasmid or plasmid expressing the indicated gene along with ISRE, IFITM3, MxA, or IRF1 promoter firefly luciferase reporter plasmid and pRL-SV40 for 24 h. Subsequently, cells were stimulated with IFN-β, IFN-γ, or IFN-λ1 (1000 U/mL) for 8 h; then, cells were harvested and cell lysates were used to determine luciferase using a Dual-Luciferase Reporter Assay System (Promega). The firefly luciferase activities were normalized to Renilla luciferase activities.

### Western blot

Cells were lysed in RIPA buffer on ice for 30 min, separated by using SDS-PAGE, and subjected to western blot analysis. Mouse monoclonal HA-tag antibody (BioLegend, cat #901515, 1:10,00), mouse monoclonal Flag-tag antibody (Sigma, cat #F3165, 1:1,000), rabbit monoclonal HA-tag antibody (CST, cat #3724, 1:1,000), rabbit monoclonal Flag-tag antibody (CST, cat #14793, 1:1,000), rabbit monoclonal myc-tag antibody (CST, cat #2278, 1:1,000), rabbit polyclonal SARS-CoV-2 S antibody (SinoBiological, cat #40591-T62, 1:5,000), and rabbit polyclonal anti-tubulin antibody (Antgene, cat #ANT327, 1:2,000) were purchased commercially. Peroxidase-conjugated secondary antibodies (Antgene, 1: 5,000) were applied accordingly, followed by image development with a Chemiluminescent HRP Substrate Kit (Millipore Corporation).

### Co-immunoprecipitation

Cells were harvested and washed with ice-cold PBS and lysed in RIPA buffer and a protease inhibitor (Roche, cat #04693116001) for 30 min on ice. The lysates were incubated with anti-HA or anti-Flag Agarose beads (MCE, cat #HY-K0207) as indicated overnight at 4°C. The immunoprecipitations were separated by SDS-PAGE and analyzed by immunoblotting.

### Immunofluorescence microscopy

HeLa cells grown on glass coverslips were fixed in 4% paraformaldehyde (in PBS) for 15 min at room temperature and permeabilized with 0.1% Triton X-100 (in PBS) for 15 min at room temperature. Subsequently, the cells were blocked with 1% BSA (in PBS) for 1 h and incubated with the rabbit monoclonal anti-HA antibody (CST, cat #3724, 1:500) or rabbit monoclonal anti-STAT1 antibody (CST, cat #14994, 1:500) or mouse monoclonal anti-Flag antibody (Sigma, cat #F3165, 1:100) overnight at 4°C. Cells were then washed with PBS and stained with secondary antibodies (Alexa Fluor R488, Invitrogen; Alexa Fluor M568, Invitrogen) for 45 min at room temperature in the dark and then washed three times with PBS. The cell nucleus was stained with DAPI (Sigma, cat #D9542) according to the standard protocols. Cell imaging was performed on a Zeiss LSM880 with an Airyscan confocal laser scanning microscope.

### Homology analysis

The full-length S protein sequences of SARS-CoV-2 (NC_045512.2 nucleotides 21563 to 25384), SARS-CoV (NC_004718.3 nucleotides 21492 to 25259), MERS-CoV (NC_019843.3 nucleotides 21456 to 25517), 229E (NC_028752.1 nucleotides 20585 to 24094), NL63 (NC_005831.2 nucleotides 20472 to 24542), and HKU-1 (NC_006577.2 nucleotides 22942 to 27012) were aligned using MUSCLE. The aligned sequences were further confirmed using a similarity plot implemented in Simplot 3.5.1.

### Statistical analysis

Data were analyzed with GraphPad Prism 8 software. Data are expressed as the mean ± SD. Comparisons of groups were performed using two two-tailed Student’s t-test or one-way ANOVA test. The values *P < 0.05, **P < 0.01, ***P < 0.001, and ****P < 0.0001 were considered significant.

## Data Availability

Source data are provided with this paper. The data that support the findings of this study are preserved at repositories of the State Key Laboratory of Virology, College of Life Sciences, Wuhan University, P.R. China, and available from the corresponding authors upon reasonable request.

## SUPPLEMENTAL MATERIAL

Fig. S1 jvi.01516-24-s0001.tif SARS-CoV-2 S protein transmembrane region did not affect the activation of ISRE and the interaction with STAT1.

Supplemental legend jvi.01516-24-s0002.docx Legend for Fig. S1.

ASM does not own the copyrights to Supplemental Material that may be linked to, or accessed through, an article. The authors have granted ASM a non-exclusive, world-wide license to publish the Supplemental Material files. Please contact the corresponding author directly for reuse.

## References

[1] Wu A, Peng Y, Huang B, Ding X, Wang X, Niu P, Meng J, Zhu Z, Zhang Z, Wang J, Sheng J, Quan L, Xia Z, Tan W, Cheng G, Jiang T. 2020. Genome composition and divergence of the novel coronavirus (2019-nCoV) originating in China. Cell Host Microbe 27:325–328. doi:10.1016/j.chom.2020.02.001 32035028 PMC7154514

[2] Lowery SA, Sariol A, Perlman S. 2021. Innate immune and inflammatory responses to SARS-CoV-2: implications for COVID-19. Cell Host Microbe 29:1052–1062. doi:10.1016/j.chom.2021.05.004 34022154 PMC8126603

[3] Kim D, Lee JY, Yang JS, Kim JW, Kim VN, Chang H. 2020. The architecture of SARS-CoV-2 transcriptome. Cell 181:914–921. doi:10.1016/j.cell.2020.04.011 32330414 PMC7179501

[4] Wu Z, McGoogan JM. 2020. Characteristics of and important lessons from the coronavirus disease 2019 (COVID-19) outbreak in China: summary of a report of 72 314 cases from the Chinese center for disease control and prevention. JAMA 323:1239–1242. doi:10.1001/jama.2020.2648 32091533

[5] Arunachalam PS, Wimmers F, Mok CKP, Perera RAPM, Scott M, Hagan T, Sigal N, Feng Y, Bristow L, Tak-Yin Tsang O, et al.. 2020. Systems biological assessment of immunity to mild versus severe COVID-19 infection in humans. Science 369:1210–1220. doi:10.1126/science.abc6261 32788292 PMC7665312

[6] Blanco-Melo D, Nilsson-Payant BE, Liu W-C, Uhl S, Hoagland D, Møller R, Jordan TX, Oishi K, Panis M, Sachs D, Wang TT, Schwartz RE, Lim JK, Albrecht RA, tenOever BR. 2020. Imbalanced host response to SARS-CoV-2 drives development of COVID-19. Cell 181:1036–1045. doi:10.1016/j.cell.2020.04.026 32416070 PMC7227586

[7] Yang D, Chu H, Hou Y, Chai Y, Shuai H, Lee A-Y, Zhang X, Wang Y, Hu B, Huang X, Yuen T-T, Cai J-P, Zhou J, Yuan S, Zhang AJ, Chan J-W, Yuen K-Y. 2020. Attenuated interferon and proinflammatory response in SARS-CoV-2-infected human dendritic cells is associated with viral antagonism of STAT1 phosphorylation. J Infect Dis 222:734–745. doi:10.1093/infdis/jiaa356 32563187 PMC7337793

[8] Salman AA, Waheed MH, Ali-Abdulsahib AA, Atwan ZW. 2021. Low type I interferon response in COVID-19 patients: interferon response may be a potential treatment for COVID-19. Biomed Rep 14:43. doi:10.3892/br.2021.1419 33786172 PMC7995242

[9] Trouillet-Assant S, Viel S, Gaymard A, Pons S, Richard J-C, Perret M, Villard M, Brengel-Pesce K, Lina B, Mezidi M, Bitker L, Belot A, COVID HCL Study group. 2020. Type I IFN immunoprofiling in COVID-19 patients. J Allergy Clin Immunol 146:206–208. doi:10.1016/j.jaci.2020.04.029 32360285 PMC7189845

[10] Hadjadj J, Yatim N, Barnabei L, Corneau A, Boussier J, Smith N, Péré H, Charbit B, Bondet V, Chenevier-Gobeaux C, et al.. 2020. Impaired type I interferon activity and inflammatory responses in severe COVID-19 patients. Science 369:718–724. doi:10.1126/science.abc6027 32661059 PMC7402632

[11] Zhang Q, Meng Y, Wang K, Zhang X, Chen W, Sheng J, Qiu Y, Diao H, Li L. 2021. Inflammation and antiviral immune response associated with severe progression of COVID-19. Front Immunol 12:631226. doi:10.3389/fimmu.2021.631226 33679778 PMC7930228

[12] Lei X, Dong X, Ma R, Wang W, Xiao X, Tian Z, Wang C, Wang Y, Li L, Ren L, Guo F, Zhao Z, Zhou Z, Xiang Z, Wang J. 2020. Activation and evasion of type I interferon responses by SARS-CoV-2. Nat Commun 11:3810. doi:10.1038/s41467-020-17665-9 32733001 PMC7392898

[13] Xia H, Cao Z, Xie X, Zhang X, Chen JY-C, Wang H, Menachery VD, Rajsbaum R, Shi P-Y. 2020. Evasion of type I interferon by SARS-CoV-2. Cell Rep 33:108234. doi:10.1016/j.celrep.2020.108234 32979938 PMC7501843

[14] Mu J, Fang Y, Yang Q, Shu T, Wang A, Huang M, Jin L, Deng F, Qiu Y, Zhou X. 2020. SARS-CoV-2 N protein antagonizes type I interferon signaling by suppressing phosphorylation and nuclear translocation of STAT1 and STAT2. Cell Discov 6:65. doi:10.1038/s41421-020-00208-3 32953130 PMC7490572

[15] Miorin L, Kehrer T, Sanchez-Aparicio MT, Zhang K, Cohen P, Patel RS, Cupic A, Makio T, Mei M, Moreno E, et al.. 2020. SARS-CoV-2 Orf6 hijacks Nup98 to block STAT nuclear import and antagonize interferon signaling. Proc Natl Acad Sci U S A 117:28344–28354. doi:10.1073/pnas.2016650117 33097660 PMC7668094

[16] Cao D, Duan L, Huang B, Xiong Y, Zhang G, Huang H. 2023. The SARS-CoV-2 papain-like protease suppresses type I interferon responses by deubiquitinating STING. Sci Signal 16:eadd0082. doi:10.1126/scisignal.add0082 37130168

[17] Wang S, Dai T, Qin Z, Pan T, Chu F, Lou L, Zhang L, Yang B, Huang H, Lu H, Zhou F. 2021. Targeting liquid-liquid phase separation of SARS-CoV-2 nucleocapsid protein promotes innate antiviral immunity by elevating MAVS activity. Nat Cell Biol 23:718–732. doi:10.1038/s41556-021-00710-0 34239064

[18] Khatun O, Sharma M, Narayan R, Tripathi S. 2023. SARS-CoV-2 ORF6 protein targets TRIM25 for proteasomal degradation to diminish K63-linked RIG-I ubiquitination and type-I interferon induction. Cell Mol Life Sci 80:364. doi:10.1007/s00018-023-05011-3 37982908 PMC11073288

[19] Müller M, Herrmann A, Fujita S, Uriu K, Kruth C, Strange A, Kolberg JE, Schneider M, Ito J, Müller MA, Drosten C, Ensser A, Sato K, Sauter D, Genotype to Phenotype Japan (G2P-Japan) Consortium. 2023. ORF3c is expressed in SARS-CoV-2-infected cells and inhibits innate sensing by targeting MAVS. EMBO Rep 24:e57137. doi:10.15252/embr.202357137 37870297 PMC10702836

[20] Schindewolf C, Lokugamage K, Vu MN, Johnson BA, Scharton D, Plante JA, Kalveram B, Crocquet-Valdes PA, Sotcheff S, Jaworski E, Alvarado RE, Debbink K, Daugherty MD, Weaver SC, Routh AL, Walker DH, Plante KS, Menachery VD. 2023. SARS-CoV-2 uses nonstructural protein 16 to evade restriction by IFIT1 and IFIT3. J Virol 97:e0153222. doi:10.1128/jvi.01532-22 36722972 PMC9973020

[21] Zhang Q, Chen Z, Huang C, Sun J, Xue M, Feng T, Pan W, Wang K, Dai J. 2021. Severe acute respiratory syndrome coronavirus 2 (SARS-CoV-2) membrane (M) and spike (S) proteins antagonize host type I interferon response. Front Cell Infect Microbiol 11:766922. doi:10.3389/fcimb.2021.766922 34950606 PMC8688923

[22] Perlman S, Dandekar AA. 2005. Immunopathogenesis of coronavirus infections: implications for SARS. Nat Rev Immunol 5:917–927. doi:10.1038/nri1732 16322745 PMC7097326

[23] Ishii KJ, Koyama S, Nakagawa A, Coban C, Akira S. 2008. Host innate immune receptors and beyond: making sense of microbial infections. Cell Host Microbe 3:352–363. doi:10.1016/j.chom.2008.05.003 18541212

[24] Tartey S, Takeuchi O. 2017. Pathogen recognition and Toll-like receptor targeted therapeutics in innate immune cells. Int Rev Immunol 36:57–73. doi:10.1080/08830185.2016.1261318 28060562

[25] Rogers NC, Slack EC, Edwards AD, Nolte MA, Schulz O, Schweighoffer E, Williams DL, Gordon S, Tybulewicz VL, Brown GD, Reis e Sousa C. 2005. Syk-dependent cytokine induction by Dectin-1 reveals a novel pattern recognition pathway for C type lectins. Immunity 22:507–517. doi:10.1016/j.immuni.2005.03.004 15845454

[26] Dosch SF, Mahajan SD, Collins AR. 2009. SARS coronavirus spike protein-induced innate immune response occurs via activation of the NF-kappaB pathway in human monocyte macrophages in vitro. Virus Res 142:19–27. doi:10.1016/j.virusres.2009.01.005 19185596 PMC2699111

[27] Mazaleuskaya L, Veltrop R, Ikpeze N, Martin-Garcia J, Navas-Martin S. 2012. Protective role of Toll-like Receptor 3-induced type I interferon in murine coronavirus infection of macrophages. Viruses 4:901–923. doi:10.3390/v4050901 22754655 PMC3386628

[28] Cai Z, Zhang MX, Tang Z, Zhang Q, Ye J, Xiong TC, Zhang ZD, Zhong B. 2020. USP22 promotes IRF3 nuclear translocation and antiviral responses by deubiquitinating the importin protein KPNA2. J Exp Med 217:e20191174. doi:10.1084/jem.20191174 32130408 PMC7201923

[29] Lukhele S, Boukhaled GM, Brooks DG. 2019. Type I interferon signaling, regulation and gene stimulation in chronic virus infection. Semin Immunol 43:101277. doi:10.1016/j.smim.2019.05.001 31155227 PMC8029807

[30] Mogensen TH. 2018. IRF and STAT transcription factors - from basic biology to roles in infection, protective immunity, and primary immunodeficiencies. Front Immunol 9:3047. doi:10.3389/fimmu.2018.03047 30671054 PMC6331453

[31] Kindler E, Thiel V, Weber F. 2016. Interaction of SARS and MERS coronaviruses with the antiviral interferon response. Adv Virus Res 96:219–243. doi:10.1016/bs.aivir.2016.08.006 27712625 PMC7112302

[32] Huang I-C, Bailey CC, Weyer JL, Radoshitzky SR, Becker MM, Chiang JJ, Brass AL, Ahmed AA, Chi X, Dong L, Longobardi LE, Boltz D, Kuhn JH, Elledge SJ, Bavari S, Denison MR, Choe H, Farzan M. 2011. Distinct patterns of IFITM-mediated restriction of filoviruses, SARS coronavirus, and influenza A virus. PLoS Pathog 7:e1001258. doi:10.1371/journal.ppat.1001258 21253575 PMC3017121

[33] Xia S, Zhu Y, Liu M, Lan Q, Xu W, Wu Y, Ying T, Liu S, Shi Z, Jiang S, Lu L. 2020. Fusion mechanism of 2019-nCoV and fusion inhibitors targeting HR1 domain in spike protein. Cell Mol Immunol 17:765–767. doi:10.1038/s41423-020-0374-2 32047258 PMC7075278

[34] Bond K, Nicholson S, Lim SM, Karapanagiotidis T, Williams E, Johnson D, Hoang T, Sia C, Purcell D, Mordant F, Lewin SR, Catton M, Subbarao K, Howden BP, Williamson DA. 2020. Evaluation of serological tests for SARS-CoV-2: implications for serology testing in a low-prevalence setting. J Infect Dis 222:1280–1288. doi:10.1093/infdis/jiaa467 32761124 PMC7454699

[35] Rengachari S, Groiss S, Devos JM, Caron E, Grandvaux N, Panne D. 2018. Structural basis of STAT2 recognition by IRF9 reveals molecular insights into ISGF3 function. Proc Natl Acad Sci U S A 115:E601–E609. doi:10.1073/pnas.1718426115 29317535 PMC5789952

[36] Khoury DS, Cromer D, Reynaldi A, Schlub TE, Wheatley AK, Juno JA, Subbarao K, Kent SJ, Triccas JA, Davenport MP. 2021. Neutralizing antibody levels are highly predictive of immune protection from symptomatic SARS-CoV-2 infection. Nat Med 27:1205–1211. doi:10.1038/s41591-021-01377-8 34002089

[37] Shrotri M, Navaratnam AMD, Nguyen V, Byrne T, Geismar C, Fragaszy E, Beale S, Fong WLE, Patel P, Kovar J, Hayward AC, Aldridge RW, Virus Watch C. 2021. Spike-antibody waning after second dose of BNT162b2 or ChAdOx1. Lancet 398:385–387. doi:10.1016/S0140-6736(21)01642-1 34274038 PMC8285117

[38] Taylor SC, Hurst B, Martiszus I, Hausman MS, Sarwat S, Schapiro JM, Rowell S, Lituev A. 2021. Semi-quantitative, high throughput analysis of SARS-CoV-2 neutralizing antibodies: measuring the level and duration of immune response antibodies post infection/vaccination. Vaccine (Auckl) 39:5688–5698. doi:10.1016/j.vaccine.2021.07.098 PMC8343386 34426026

[39] Shu YJ, He JF, Pei RJ, He P, Huang ZH, Chen SM, Ou ZQ, Deng JL, Zeng PY, Zhou J, Min YQ, Deng F, Peng H, Zhang Z, Wang B, Xu ZH, Guan WX, Hu ZY, Zhang JK. 2021. Immunogenicity and safety of a recombinant fusion protein vaccine (V-01) against coronavirus disease 2019 in healthy adults: a randomized, double-blind, placebo-controlled, phase II trial. Chin Med J (Engl) 134:1967–1976. doi:10.1097/CM9.0000000000001702 34310400 PMC8382383

[40] Achiron A, Mandel M, Dreyer-Alster S, Harari G, Gurevich M. 2021. Humoral SARS-COV-2 IgG decay within 6 months in COVID-19 healthy vaccinees: the need for a booster vaccine dose? Eur J Intern Med 94:105–107. doi:10.1016/j.ejim.2021.10.027 34742628 PMC8549192

[41] Aldridge RW, Yavlinsky A, Nguyen V, Eyre MT, Shrotri M, Navaratnam AMD, Beale S, Braithwaite I, Byrne T, Kovar J, Fragaszy E, Fong WLE, Geismar C, Patel P, Rodger A, Johnson AM, Hayward A. 2022. SARS-CoV-2 antibodies and breakthrough infections in the Virus Watch cohort. Nat Commun 13:4869. doi:10.1038/s41467-022-32265-5 35982056 PMC9387883

[42] Amanna IJ, Carlson NE, Slifka MK. 2007. Duration of humoral immunity to common viral and vaccine antigens. N Engl J Med 357:1903–1915. doi:10.1056/NEJMoa066092 17989383

[43] Antia A, Ahmed H, Handel A, Carlson NE, Amanna IJ, Antia R, Slifka M. 2018. Heterogeneity and longevity of antibody memory to viruses and vaccines. PLoS Biol 16:e2006601. doi:10.1371/journal.pbio.2006601 30096134 PMC6105026

[44] Deng J, Zheng SN, Xiao Y, Nan ML, Zhang J, Han L, Zheng Y, Yu Y, Ding Q, Gao C, Wang PH. 2023. SARS-CoV-2 NSP8 suppresses type I and III IFN responses by modulating the RIG-I/MDA5, TRIF, and STING signaling pathways. J Med Virol 95:e28680. doi:10.1002/jmv.28680 36929724

[45] Deng J, Zheng Y, Zheng SN, Nan ML, Han L, Zhang J, Jin Y, Pan JA, Gao C, Wang PH. 2023. SARS-CoV-2 NSP7 inhibits type I and III IFN production by targeting the RIG-I/MDA5, TRIF, and STING signaling pathways. J Med Virol 95:e28561. doi:10.1002/jmv.28561 36755358

[46] Wu Y, Ma L, Zhuang Z, Cai S, Zhao Z, Zhou L, Zhang J, Wang PH, Zhao J, Cui J. 2020. Main protease of SARS-CoV-2 serves as a bifunctional molecule in restricting type I interferon antiviral signaling. Signal Transduct Target Ther 5:221. doi:10.1038/s41392-020-00332-2 33024073 PMC7537955

[47] Dai L, Gao GF. 2021. Viral targets for vaccines against COVID-19. Nat Rev Immunol 21:73–82. doi:10.1038/s41577-020-00480-0 33340022 PMC7747004

[48] Yan R, Zhang Y, Li Y, Xia L, Guo Y, Zhou Q. 2020. Structural basis for the recognition of SARS-CoV-2 by full-length human ACE2. Science 367:1444–1448. doi:10.1126/science.abb2762 32132184 PMC7164635

[49] Walls AC, Park YJ, Tortorici MA, Wall A, McGuire AT, Veesler D. 2020. Structure, function, and antigenicity of the SARS-CoV-2 spike glycoprotein. Cell 181:281–292. doi:10.1016/j.cell.2020.02.058 32155444 PMC7102599

[50] Coutard B, Valle C, de Lamballerie X, Canard B, Seidah NG, Decroly E. 2020. The spike glycoprotein of the new coronavirus 2019-nCoV contains a furin-like cleavage site absent in CoV of the same clade. Antiviral Res 176:104742. doi:10.1016/j.antiviral.2020.104742 32057769 PMC7114094

[51] Forni D, Cagliani R, Clerici M, Sironi M. 2017. Molecular evolution of human coronavirus genomes. Trends Microbiol 25:35–48. doi:10.1016/j.tim.2016.09.001 27743750 PMC7111218

[52] Kirby T. 2021. New variant of SARS-CoV-2 in UK causes surge of COVID-19. Lancet Respir Med 9:e20–e21. doi:10.1016/S2213-2600(21)00005-9 33417829 PMC7784534

[53] Cele S, Jackson L, Khoury DS, Khan K, Moyo-Gwete T, Tegally H, San JE, Cromer D, Scheepers C, Amoako D, et al.. 2021. SARS-CoV-2 Omicron has extensive but incomplete escape of Pfizer BNT162b2 elicited neutralization and requires ACE2 for infection. medRxiv:2021.12.08.21267417. doi:10.1101/2021.12.08.21267417

[54] Lokugamage KG, Hage A, de Vries M, Valero-Jimenez AM, Schindewolf C, Dittmann M, Rajsbaum R, Menachery VD. 2020. Type I interferon susceptibility distinguishes SARS-CoV-2 from SARS-CoV. J Virol 94(23):e01410-20. doi:10.1128/JVI.01410-20 PMC7654262 32938761

[55] Rahmani H, Davoudi-Monfared E, Nourian A, Khalili H, Hajizadeh N, Jalalabadi NZ, Fazeli MR, Ghazaeian M, Yekaninejad MS. 2020. Interferon β-1b in treatment of severe COVID-19: a randomized clinical trial. Int Immunopharmacol 88:106903. doi:10.1016/j.intimp.2020.106903 32862111 PMC7445008

[56] Alavi Darazam I, Shokouhi S, Pourhoseingholi MA, Naghibi Irvani SS, Mokhtari M, Shabani M, Amirdosara M, Torabinavid P, Golmohammadi M, Hashemi S, Azimi A, Jafarazadeh Maivan MH, Rezaei O, Zali A, Hajiesmaeili M, Shabanpour Dehbsneh H, Hoseyni Kusha A, Taleb Shoushtari M, Khalili N, Soleymaninia A, Gachkar L, Khoshkar A. 2021. Role of interferon therapy in severe COVID-19: the COVIFERON randomized controlled trial. Sci Rep 11:8059. doi:10.1038/s41598-021-86859-y 33850184 PMC8044200

[57] Zhang Z, Zhou L, Liu Q, Zheng Y, Tan X, Huang Z, Guo M, Wang X, Chen X, Liang S, Li W, Song K, Yan K, Li J, Li Q, Zhang Y, Yang S, Cai Z, Dai M, Xian Q, Shi ZL, Xu K, Lan K, Chen Y. 2024. The lethal K18-hACE2 knock-in mouse model mimicking the severe pneumonia of COVID-19 is practicable for antiviral development. Emerg Microbes Infect 13:2353302. doi:10.1080/22221751.2024.2353302 38753462 PMC11132709

[58] Platanitis E, Demiroz D, Schneller A, Fischer K, Capelle C, Hartl M, Gossenreiter T, Müller M, Novatchkova M, Decker T. 2019. A molecular switch from STAT2-IRF9 to ISGF3 underlies interferon-induced gene transcription. Nat Commun 10:2921. doi:10.1038/s41467-019-10970-y 31266943 PMC6606597

[59] Shi J, Du T, Wang J, Tang C, Lei M, Yu W, Yang Y, Ma Y, Huang P, Chen H, Wang X, Sun J, Wang H, Zhang Y, Luo F, Huang Q, Li B, Lu S, Hu Y, Peng X. 2023. Aryl hydrocarbon receptor is a proviral host factor and a candidate pan-SARS-CoV-2 therapeutic target. Sci Adv 9:eadf0211. doi:10.1126/sciadv.adf0211 37256962 PMC10413656

[60] Martinez-Moczygemba M, Gutch MJ, French DL, Reich NC. 1997. Distinct STAT structure promotes interaction of STAT2 with the p48 subunit of the interferon-alpha-stimulated transcription factor ISGF3. J Biol Chem 272:20070–20076. doi:10.1074/jbc.272.32.20070 9242679

[61] Nan J, Wang Y, Yang J, Stark GR. 2018. IRF9 and unphosphorylated STAT2 cooperate with NF-κB to drive IL6 expression. Proc Natl Acad Sci U S A 115:3906–3911. doi:10.1073/pnas.1714102115 29581268 PMC5899435

[62] Mariani MK, Dasmeh P, Fortin A, Kalamujic M, Caron E, Harrison AN, Cervantes-Ortiz S, Mukawera E, Serohijos AWR, Grandvaux N. 2018. RNASeq analysis identifies non-canonical role of STAT2 and IRF9 in the regulation of a STAT1-independent antiviral and immunoregulatory transcriptional program induced by IFNβ and TNFα. bioRxiv. doi:10.1101/273623

