# Supplementary figures and images for: SARS-CoV-2 S protein disrupts the formation of ISGF3 complex through conserved S2 subunit to antagonize type I interferon response
Source: J Virol. 2024 Dec 19;99(1):e01516-24. doi: 10.1128/jvi.01516-24 (PMC11784297; doi:10.1128/jvi.01516-24)

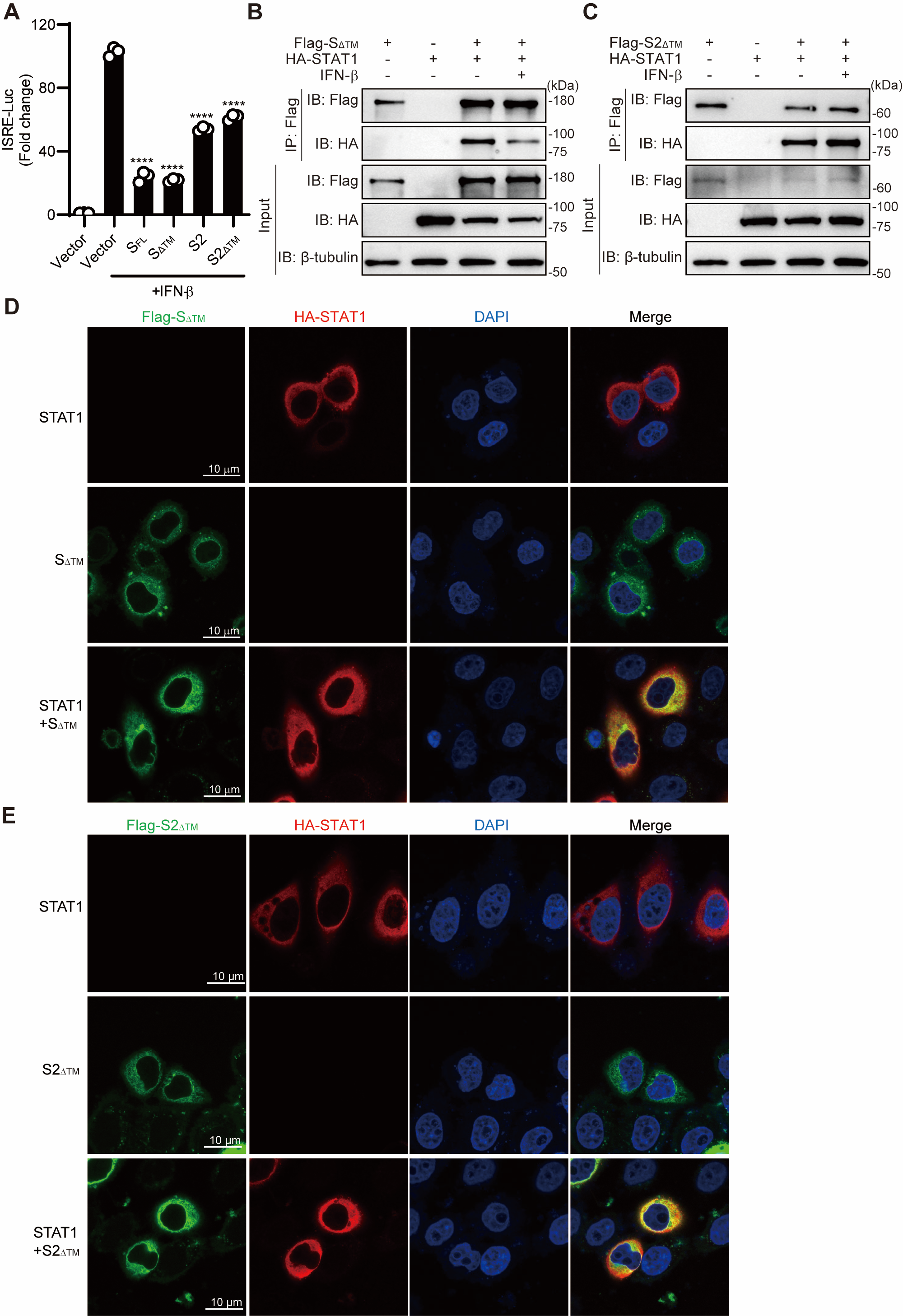

Supplement: Fig. S1 — SARS-CoV-2 S protein transmembrane region did not affect the activation of ISRE and the interaction with STAT1. [file jvi.01516-24-s0001.tif]
