# Supplementary material for: SARS-CoV-2 S protein disrupts the formation of ISGF3 complex through conserved S2 subunit to antagonize type I interferon response
Source: J Virol. 2024 Dec 19;99(1):e01516-24. doi: 10.1128/jvi.01516-24 (PMC11784297; doi:10.1128/jvi.01516-24)
Supplement: Supplemental legend — Legend for Fig. S1. [file jvi.01516-24-s0002.docx]

**Supplemental Fig. 1. SARS-CoV-2 S protein transmembrane region did not affect the activation of ISRE and the interaction with STAT1.**

(A) HEK293T cells were co-transfected with pISRE-Luc, Renilla luciferase control plasmid pRL-SV40, and S protein- and its truncations- expressing plasmids. 24 h after the initial transfection, the cells were stimulated with IFN-β (1,000 U/mL) for 12 h, and the luciferase assays were measured. (B, C) HEK293 cells were transfected with plasmid expression HA-STAT1 along with Flag-S_ΔTM_ or Flag-S2_ΔTM_ for 24 h, followed by Co-Immunoprecipitation and Western Blot analysis. (D, E) HeLa cells were transfected with plasmid expression HA-STAT1 along with Flag-S_ΔTM_ or Flag-S2_ΔTM_ for 24 h. Cells were fixed and permeabilized, staining with anti-HA and anti-FLAG as primary antibodies, and anti-Alexa Fluor 488, and anti-Alexa Fluor 568 as secondary antibodies. Scale bar, 10 μm. Error bars represent mean ± SD from three independent experiments. Statistical significance was determined by the One-Way ANOVA test. ****P < 0.0001.
